# Deductions from Ferran’s Inoculation Statistics

**Published:** 1887-02

**Authors:** J. E. Winans

**Affiliations:** Lyons Farms, New Jersey


### DEDUCTIONS FROM FERRAN'S INOCULATION STATISTICS FOUND ON PAGE 22, JANUARY NO. OF THE HOMŒOPATHIC PHYSICIAN.

J. E. Winans, M. D., Lyons Farms, New Jersey.

(*a*) *Number inoculated* were in proportion to those *non-inoculated* as three in seventeen.

(*b*) While *proportion attacked* were relatively less than three in one hundred, as against seven in one hundred.

(*c*) Yet *actual number of deaths* slightly preponderated (proportionately) among the *inoculated*, viz.: seven in thirty-four, or about one to five, which somewhat exceeds above ratio of three to seventeen.

(*d*) While as to *actual fatality* among persons attacked there were *nearly eighteen per cent.* of the *inoculated*, as against *six per cent.* of those *non-inoculated.* Ergo, hurrah for microbic inoculations!

